# New uracil analog as inhibitor/modulator of ABC transporters or/and NF-κB in taxol-resistant MCF-7/Tx cell line

**DOI:** 10.1007/s00432-024-05833-z

**Published:** 2024-06-25

**Authors:** Angelika Długosz-Pokorska, Tomasz Janecki, Anna Janecka, Katarzyna Gach-Janczak

**Affiliations:** 1 Department of Biomolecular Chemistry, Medical University of Lodz, Mazowiecka 6/8, 92-215 Lodz, Poland; 2 Institute of Organic Chemistry, Faculty of Chemistry, Lodz University of Technology, Lodz, Poland

**Keywords:** ABC transporters, MDR, Taxol, Tx-resistant modulator

## Abstract

**Purpose:**

The global increase in breast cancer cases necessitates ongoing exploration of advanced therapies. Taxol (Tx), an initial breast cancer treatment, induces mitotic arrest but faces limitations due to side effects and the development of resistance. Addressing Tx resistance involves understanding the complex molecular mechanisms, including alterations in tubulin dynamics, NF-κB signaling, and overexpression of ABC transporters (ABCB1 and ABCG2), leading to multidrug resistance (MDR).

**Methods:**

Real-time PCR and ELISA kits were used to analyze ABCB1, ABCG2 and NF-κB gene and protein expression levels, respectively. An MDR test assessed the resistance cell phenotype.

**Results:**

MCF-7/Tx cells exhibited a 24-fold higher resistance to Tx. Real-time PCR and ELISA analysis revealed the upregulation of ABCB1, ABCG2, and NF-κB. U-359 significantly downregulated both ABCB1 and ABCG2 gene and protein levels. Co-incubation with Tx and U-359 further decreased the mRNA and protein expression of these transporters. The MDR test indicated that U-359 increased MDR dye retention, suggesting its potential as an MDR inhibitor. U-359 and Tx, either individually or combined, modulated NF-κBp65 protein levels.

**Conclusion:**

The development of a Taxol-resistant MCF-7 cell line provided valuable insights. U-359 demonstrated effectiveness in reducing the expression of ABC transporters and NF-κB, suggesting a potential solution for overcoming multidrug resistance in breast cancer cells. The study recommends a strategy to enhance the sensitivity of cancer cells to chemotherapy by integrating U-359 with traditional drugs.

## Introduction

Breast cancer is a persistent global health challenge, characterized by a consistent rise in incidence rates (Sung et al. 2021). There are several surgical procedures combined with pharmacotherapy options for treating breast cancer patients. Despite the advancement in breast cancer treatment, the survival rates after relapse are still not satisfactory, emphasizing the need to explore innovative and more effective chemotherapeutic alternatives.

Chemotherapeutics used in breast cancer treatment belong to different drug categories, including alkylating agents (e.g., cyclophosphamide), antimetabolites (e.g., methotrexate), anticancer antibiotics (e.g., doxorubicin), and antimitotic agents (e.g., Taxol) (Gallego-Jara et al. 2020; Haggag et al. 2022, 2021; Wani et al. 1971). Among these, Taxol (Tx) has emerged as one of the most effective drugs in the initial treatment of breast cancer. At the molecular level, Tx is an antimitotic agent that binds microtubules inhibiting their disassembly (Janse van Vuuren et al. 2015). This interaction leads to the arrest of mitosis, inhibition of the cell cycle, and ultimately induces apoptosis, resulting in the death of numerous cancer cells (Gallego-Jara et al. 2020; Janse van Vuuren et al. 2015; Lim et al. 2022). Despite its initial success, the clinical utility of Tx is limited by severe side effects and the development of resistance (Zajdel et al. 2019).

Overcoming Tx resistance requires a comprehensive understanding of complex molecular mechanisms contributing to the multidrug resistance phenotype. Recent research has shed light on various pathways through which cancer cells acquire resistance to Tx, including alterations in tubulin dynamics, modulation of nuclear factor κB (NF-κB), and overexpression of ATP-binding cassette (ABC) transporters (Długosz-Pokorska et al. 2023; Němcová-Fürstová et al. 2016; Murray et al. 2012).

The ABC transporter family, comprising a diverse group of membrane proteins, plays an important role in cellular drug efflux and is implicated in the development of multiple drug resistance (MDR) (Długosz and Janecka 2016). ABC transporters such as P-glycoprotein (ABCB1) and breast cancer resistance protein (ABCG2) actively extrude chemotherapeutic agents like Tx from cancer cells, thereby reducing their intracellular concentrations and contributing to resistance (Zajdel et al. 2019). The regulation of these transporters is closely correlated with the activity of transcription factors, such as NF-κB/p65, which exert control over the expression of genes involved in Tx efflux and cellular survival (Mosca et al. 2021). The NF-κB family is a transcription factor protein complexes that controls transcription of DNA, cytokine production, and cell survival. The most important subunit of the NF-κB family is RelA/p65, which is phosphorylated in the posttranslational activation mechanism (Schmitz and Baeuerle 1991; Huang et al. 2010). The genes whose expression is regulated by NF-κB RelA/p65 play an important role in immune/stress responses, apoptosis, proliferation, differentiation, and development (Fahy et al. 2004; Sakamoto et al. 2009).

In our previous study, searching for novel anticancer agents, we investigated a series of uracil analogs characterized by an exo-cyclic methylidene group linked with a carbonyl moiety (Pięta et al. 2019). Among these analogs, 3-p-bromophenyl-1-ethyl-5-methylidenedihydrouracil (U-359) emerged as the most potent, demonstrating significant inhibition of MCF-7 cells' proliferation and inducing apoptosis via the mitochondrial pathway. Moreover, U-359 displayed a synergistic anticancer effect with Tx and reversed resistance of MCF-7 cells to Tx (Długosz-Pokorska et al. 2023). In the same study, we investigated the possible mechanism responsible for Tx resistance development in MCF-7 cells and assessed the expression levels of key proteins involved in microtubule dynamics, including tubulin III (TUBIII) and Nlp. Our experiments revealed the significant reduction in the levels of Nlp and TUBIII after co-administration of Tx and U-359, contrasting with the effects of Tx alone. It was concluded that U-359 can overcome Tx resistance in MCF-7 cells by modulating microtubule dynamics and stabilizing microtubules (Długosz-Pokorska et al. 2023). These research findings hold significant implications for the advancement of more effective strategies in breast cancer treatment (Długosz-Pokorska et al. 2020, 2023; Pięta et al. 2019)The aim of this study was to investigate the other mechanisms involved in Tx resistant development. Therefore, the gene and protein expression profiles of ABC transporters and NF-kB in both MCF-7 and MCF-7 resistance to Tx (MCF-7/Tx) cell lines were investigated. Additionally, we tested the potential of U-359 as an inhibitor of ABC transporters and/or NF-kB activation in MCF-7 and MCF-7/Tx cell lines, alone and in combination with Tx.

## Materials and methods

### Materials

The uracil analog U-359 was obtained according to the Horner-Wadsworth-Emmons methodology published earlier (Pięta et al. 2019). Tx was purchased from Sigma-Aldrich (St. Louis, MO, USA). For all experimental procedures, U-359 and Tx were solubilized in dimethyl sulfoxide (DMSO) and subsequently diluted in culture medium to achieve a DMSO concentration of less than 0.1%.

### Cell culture

The MCF-7 breast cancer cell line, which is positive for hormone receptors (estrogen receptor, ER and progesterone receptor, PR), was purchased from the European Collection of Cell Cultures (ECACC). The cells were cultured in Minimum Essential Medium Eagle (MEME) supplemented with non-essential amino acids and antibiotics (streptomycin at 100 mg/mL and penicillin at 100 U/mL), along with 2 mM glutamine. All these materials were obtained from Sigma Aldrich, St. Louis, MO, USA. Additionally, 10% fetal bovine serum from Biological Industries, Beit-HaEmek, Israel, was added to the culture medium. The cells were maintained at 37 °C in a 5% CO_2_ atmosphere and allowed to grow until they reached 80% confluence.

### Selection of Tx-resistant MCF-7 cells (MCF-7/Tx)

For the selection of Tx-resistant cells, MCF-7 cells underwent prolonged exposure to progressively increasing concentrations of Tx, ranging from 0.01 μM to 1 μM. The viability and proliferation of these cells were subsequently evaluated using the MTT assay. If their growth pattern closely resembled that of the original MCF-7 cells, the Tx concentration was further increased. This iterative process continued until the cells developed tolerance to a maximum Taxol concentration of 1 μM. Throughout all experimental procedures, the Taxol-resistant MCF-7 cells were consistently designated as MCF-7/Tx.

#### Determination of cell morphology using light microscopy

To determine morphological alterations in MCF-7 and MCF-7/Tx cells, we used Wright and Giemsa staining, sourced from Merck, Germany. Giemsa Dye was applied to impart varying colorations to the cytoplasm, ranging from orange to pink, while the nucleus was dyed in tones of blue to purple, contingent upon the cytoplasmic acidity.

In brief, the protocol initiated with the seeding of cells onto 6-well plates at a density of 4 × 10^5^ cells per well. Subsequently, these cells underwent a 24-h incubation with U-359 and/or Tx at their respective IC_50_ concentrations, using untreated cells as a negative control. Following the incubation period, the medium was aspirated, and the cells were fixed in methanol for 5 min. Post-fixation, PBS was used for cell washing, followed by staining with Wright and Giemsa solution for 30 min. Subsequent to staining, the cells were subjected to two rinses with H_2_O and left to air-dry. Finally, the stained cells were photographed using a light microscope.

### MTT-assay

The MTT assay was conducted following the established protocol by Mosmann (1983). In summary, cells (10^4^ per well) were plated in 24-well plates with 100 µl of medium and allowed to grow. After 24 h, the cells were exposed to varying concentrations of the compounds under investigation, achieving final concentrations ranging from 0 to 100 μM, for an additional 24 h. Subsequently, the cells were incubated for 2 h at 37 °C with MTT solution [5 mg/ml in phosphate-buffered saline (PBS, Gibco, Invitrogen, Carlsbad, CA, USA)]. The resulting blue formazan product's absorbance was measured at 560 nm using an iMark Bio-Rad microplate reader (Hercules, CA, USA) and compared to the control (untreated cells). The IC_50_ values (the concentration at which 50% inhibition occurs) were then determined.

### Quantitative real-time PCR assay

Real-time quantitative PCR (RT-qPCR) was used to assess the mRNA levels of ABC transporter and NF-κB genes in MCF-7 and MCF-7/Tx cells. Initially, MCF-7 and MCF-7/Tx cells were seeded onto 6-well plates at a cell density of 5.0 × 10^5 cells per well and left to grow. Following this, the cells were incubated with U-359, Tx, or U-359 + Tx at their respective IC_50_ concentrations for 24 h. The effects of the combination treatment were then compared with those observed when Tx was administered individually. To obtain total RNA, the Total RNA Mini Kit (A&A Biotechnology, Gdynia, Poland) was employed, following the manufacturer's protocol. The concentration of RNA was determined using a Nanophotometer, and a consistent concentration of 150 ng/µL was used for subsequent experiments. cDNA synthesis was carried out using the Transcriba Kit (A&A Biotechnology, Gdynia, Poland). Gene-specific primers (*ABCB1, ABCG2*, and *NF-κB/p65*) listed in Table 1 were used for amplification. Real-Time 2x-PCR SYBR Master Mix (A&A Biotechnology, Gdynia, Poland) was utilized for this purpose in the Stratagene MX3005P QPCR System (Agilent Technologies, Inc., Santa Clara, CA, USA), following the manufacturer's instructions. GAPDH was employed as the internal reference gene to standardize the expression of the investigated genes. The expression levels of the tested genes were determined using the 2^-∆∆CT^ method (Winer et al. 1999).

**Table 1:** Primer sequences for real-time PCR reaction

Gene	Primer sequences	
	Forward primer	Reverse primer
***GAPDH***	GTCGCTGTTGAAGTCAGAGGAG	CGTGTCAGTGGTGGACCTGAC
***NF-κB/p65***	TGCCAACAGATGGCCCATAC	TGTTCTTTTCACTAGAGGCACCA
***ABCB1***	GTGGGGCAAGTCAGTTCATT	TCTTCACCTCCAGGCTCAGT
***ABCG2***	GCTTTCTACCTGCACGAAAACCAGTTGAG	ATGGCGTTGAGACCAG

### Assessment of ABCB1 and ABCG2 protein levels by ELISA-based method

The protein levels of ABCB1 and ABCG2 in MCF-7 and MCF-7/Tx cells were determined using an ELISA-based method, including ABCB1 (Multidrug resistance protein 1 kit) and ABCG2 (ATP-binding cassette sub-family G member 2 kit), provided by Finetest BT LAB (Shanghai, China).

Briefly, MCF-7 and MCF-7/Tx cells were seeded on 6-well plates at the density of 5.0 × 10^5^ cells per well and left to grow for 24 h. Subsequently, the cells were incubated with U-359, Tx, or U-359 + Tx at their respective IC_50_ concentrations for 24 h. Following the incubation period, the cells were washed with PBS and harvested by centrifugation at 200xg for 5 min. Cell lysates were prepared following the provided protocol. For the ELISA assay, appropriately diluted protein extracts (50 μg) and standards were added to the wells of 96-well plates pre-coated with specific antibodies for ABCB1 and ABCG2. The specific ABC transporter proteins in the tested samples are bound to the immobilized antibodies on the plate surface. A secondary antibody, conjugated with horseradish peroxidase (HRP), was then added. This HRP conjugate enabled a sensitive colorimetric readout. Finally, a stop solution was added to each well, and the optical density (OD) of the resulting yellow solution was measured spectrometrically at a wavelength of 450 nm to quantify the protein levels of ABCB1 and ABCG2 in the samples.

### Multidrug resistance assay kit

To detect the multidrug-resistant phenotype in breast cancer cells, we used the Multidrug Resistance Assay Kit from Sigma-Aldrich (St. Louis, USA) and followed the manufacturer's guidelines.

Briefly, MCF-7 and MCF-7/Tx cells were seeded in 96-well plates at a density of 8.0 × 10^4^ cells per well and left to grow for 24 h. The cells were incubated with U-359, Tx, or U-359 + Tx at their respective IC_50_ concentrations for 24 h. Subsequently, 100 μL of the MDR Dye Loading Solution was added to each well. The plates were then incubated at 37 °C in a humidified environment with 5% CO_2_ for a duration of 15 min. The analysis of fluorescence intensity was carried out using a Flexstation 3 instrument.

### Assessment of NF-κB protein level by ELISA-based method

NF-κB/p65 activity was assessed in cellular protein extracts (10 μg) using the Human Nuclear factor NF-kappa-B p65 subunit ELISA Kit (BT LAB, Shanghai, China).

Briefly, MCF-7 and MCF-7/Tx cells were seeded in 6-well plates at a density of 5.0 × 10^5^ cells per well and left to grow for 24 h. The cells were then incubated with U-359 (specific inhibitor), Tx (test substance), or a combination of U-359 and Tx at their respective IC_50_ concentrations for 24 h. Subsequently, the cells were washed with PBS and collected by centrifugation (200xg, 5 min), followed by the collection of cell culture supernatants.

Nuclear extracts were obtained through repeated freeze–thaw cycles to release the intracellular components. These extracts were then analyzed using a kit comprising a 96-well plate with immobilized oligonucleotides for the NF-κB subunit. NF-κB present in the sample bound to antibodies coated on the wells. Biotinylated human NF-κB antibody was added, binding to NF-κB in the sample. Subsequently, streptavidin-HRP was added, binding to the biotinylated NF-κB antibodies. After a washing step to remove unbound streptavidin-HRP, substrate solution was added, resulting in color development proportional to the amount of NF-κB. The reaction was terminated by the addition of acidic stop solution, and absorbance was measured at 450 nm.

### Statistical analysis

Statistical analysis was performed using Prism 6.0 software (GraphPad Software Inc., San Diego, CA, USA). The study collected data from at least three independent experiments, each conducted in triplicate, and the results were expressed as the mean ± standard error of the mean (SEM). To determine the statistical significance, a one-way analysis of variance (ANOVA) was initially applied, followed by a post-hoc multiple comparison Student–Newman–Keuls test or test *T*. Levels of significance were denoted as follows: *p < 0.05, **p < 0.01, and ***p < 0.001 (compared with control) or ^#^*p* < 0.05, ^##^*p* < 0.01, and ^###^*p* < 0.001 (compared with Tx) indicating the statistical significance of the findings.

## Results

### MCF-7/Tx cells, derived from MCF-7 cells exposed to increasing Tx concentrations, exhibited morphological changes, increased proliferation, and drug resistance.

**Fig. 1:**
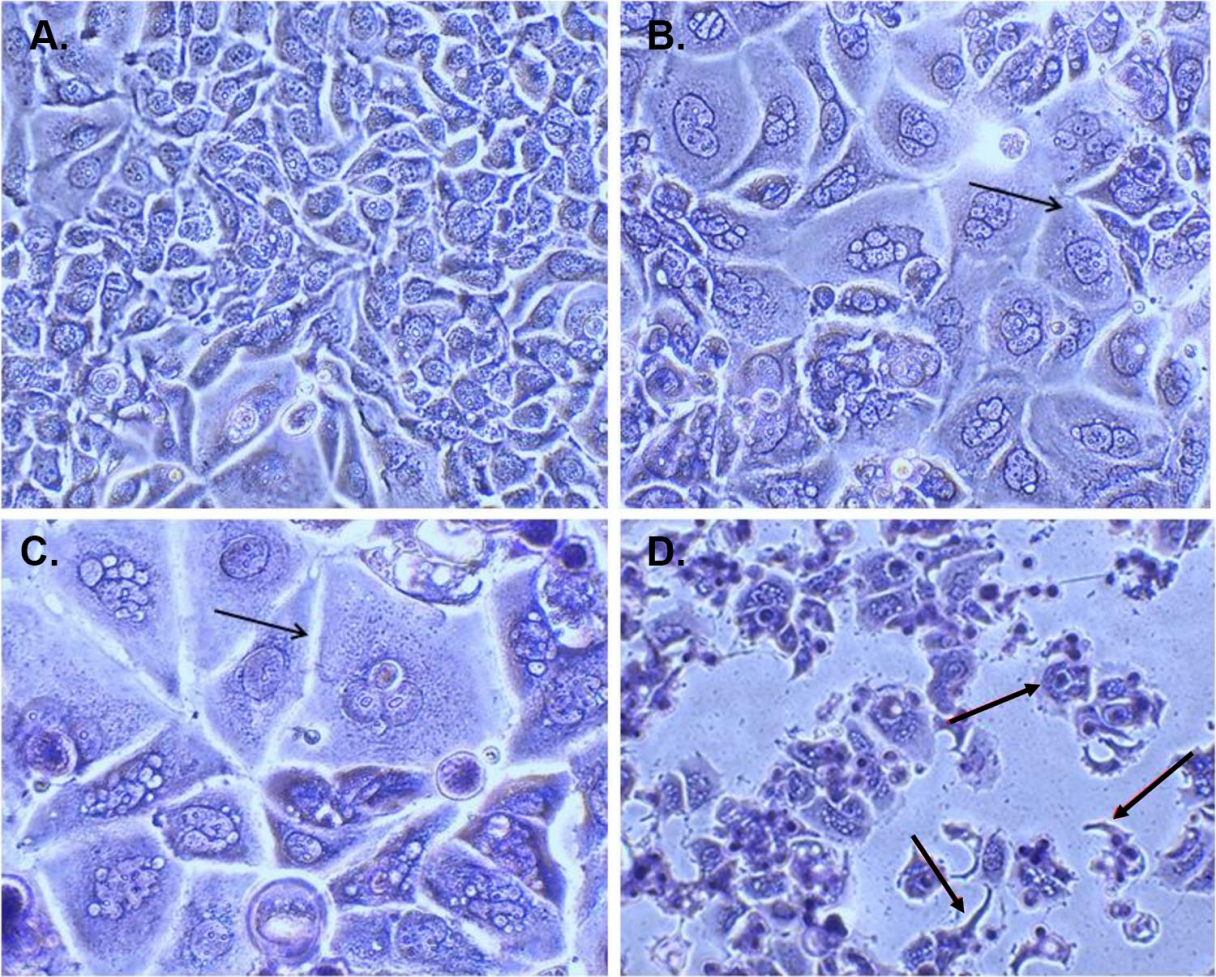
Morphological characterization of MCF-7 and MCF-7 Tx-resistant cells investigated through light microscopy. Cells were stained using Wright-Giemsa stain (original magnification, x1,000). (**A**) Sensitive MCF-7 cells; (**B**) MCF-7/Tx cells; (**C**) MCF-7/Tx cells treated with Tx at the IC_50_ concentration for MCF-7 (0.04 μM) for 24 hours, by black arrows. The examples of changes cells are indicated by black arrows. (**D**) MCF-7/Tx cells treated with Tx at the IC_50_ concentration for resistant cells (0.96 μM) for 24 hours, marked with black arrows, showed chromatin condensation, cytoplasmic changes, and the presence of apoptotic bodies

MCF-7/Tx cells were derived from MCF-7 cells by gradually exposing them to increasing concentrations of Tx, ranging from IC_10_ to IC_50_ levels, in a stepwise manner over time. This prolonged exposure continued until the cells developed tolerance to the drug and could proliferate normally, with 50% of the population surviving at a concentration of 0.96 μM. The development of drug-resistant characteristics in these cells is typically linked to various biological changes, including alterations in cell morphology (Song et al. 2023). Unlike the original MCF-7 cells, MCF-7/Tx cells became larger, exhibited an irregular and rounded shape with poorly defined edges, and contained numerous nucleoli within the cytoplasm. When treated with Tx, MCF-7/Tx cells showed a more elongated morphology compared to untreated cells, yet maintained membrane integrity, as illustrated in Fig. 1. Furthermore, MCF-7/Tx cells demonstrated a significantly higher proliferation rate than MCF-7 cells.

### Taxol-induced morphological and proliferative changes in MCF-7/Tx Cells

**Fig. 2:**
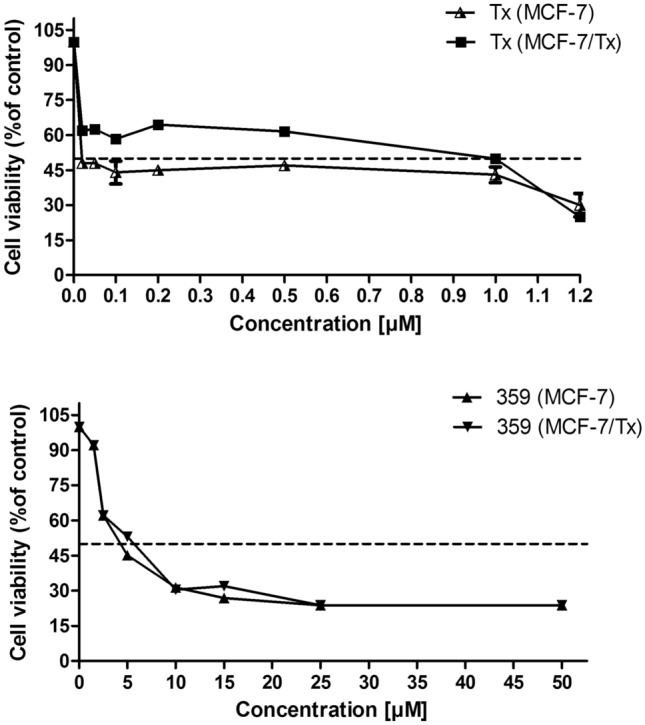
The cytotoxic effect of Tx and 359 in MCF-7 and MCF-7/Tx cells analyzed by MTT assay. Each data point represents the mean of three replicates, and the error bars indicate the standard deviation

The cytotoxicity of Tx or U-359 in MCF-7 and MCF-7/Tx cells was assessed using the MTT assay. The IC_50_ values obtained were 0.04 μM and 0.96 μM for MCF-7 and MCF-7/Tx cells respectively. To gauge resistance, the resistance index (R) was calculated as R = IC_50_ resistant cells/IC_50_ sensitive cells. Consequently, MCF-7/Tx cells exhibited approximately 24-fold higher resistance to Tx compared to the parental cell line. Conversely, the IC_50_ values for U-359 were approximately 3.6 μM for both cancer cell lines (Fig. 2).

### Taxol upregulated genes, notably in MCF-7/Tx cells, while U-359, especially in combination with taxol, downregulated them

The expression levels of *ABCB1, ABCG2* and *NF-κB* genes in both MCF-7 and MCF-7/Tx cells were analyzed using real-time PCR.

**Fig. 3:**
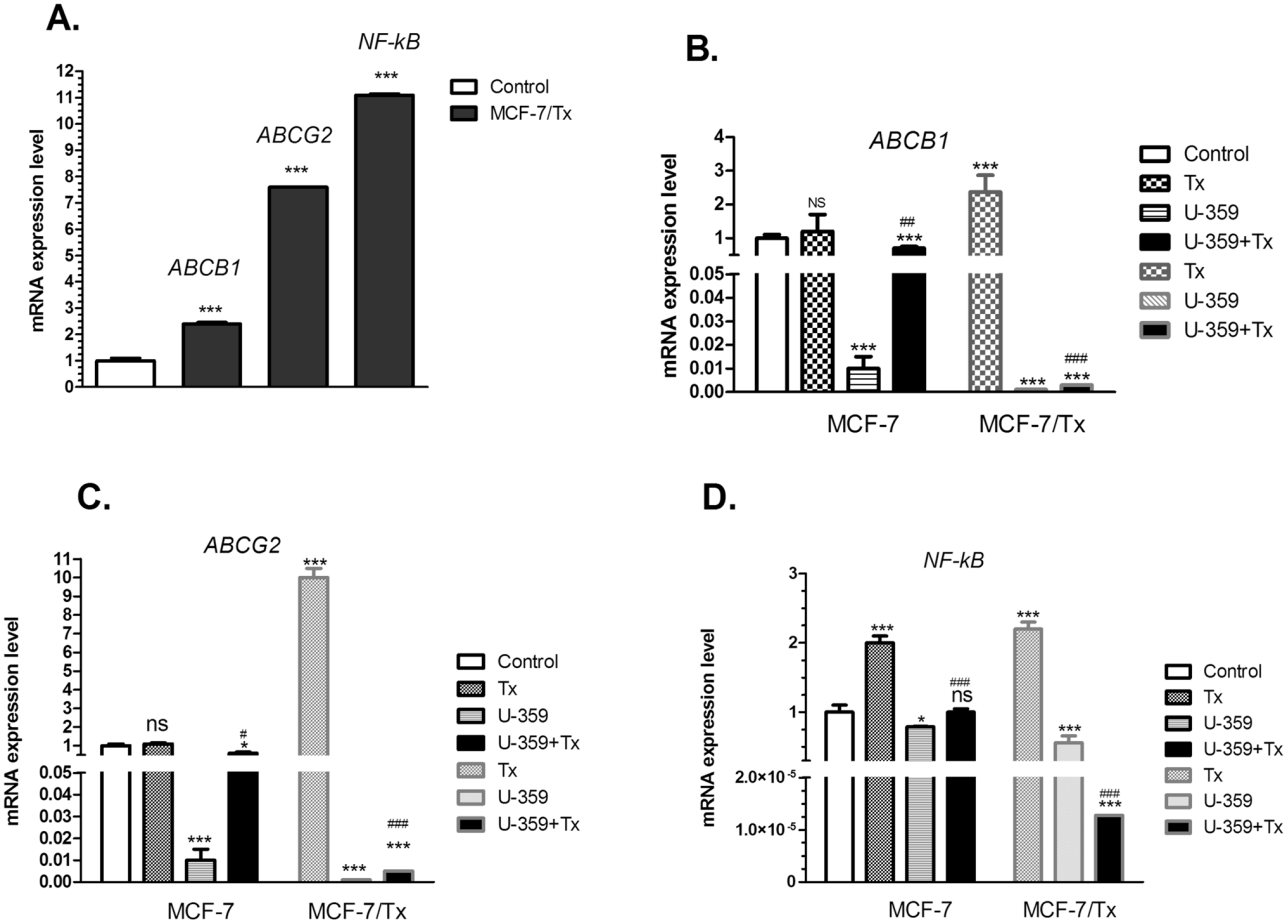
Real-time PCR analysis of mRNA levels of ABCB1, ABCG2, and NF-κB in MCF-7/Tx cells compared to parental MCF-7 cells (**A**). The effect of Tx, U-359, or a combination of U-359 and Tx treatment on mRNA expression levels of ABCB1 (**B**), ABCG2 (**C**), and NF-κB (**D**). Data are presented as mean ± SEM. In each experiment, three replicates were used. Statistical significance was evaluated using one-way ANOVA followed by a post-hoc multiple comparison Student–Newman–Keuls test. ^*^p<0.05, ^***^p < 0.001, compared with control; ^#^*p* < 0.05, ^##^*p* < 0.01, ^###^*p* < 0.001 compared with Tx

In MCF-7/Tx cells, we observed the notable increase in the relative mRNA expression levels of *ABCB1* (1.4-fold), *ABCG2* (6.6-fold) and *NF-κB* (10.1-fold), in comparison to the parental MCF-7 cells (Fig. 3A). The exposure of MCF-7/Tx cells to Tx resulted in a significant up-regulation of tested genes *ABCB1, ABCG2* and *NF-κB* of 2.4-fold, tenfold, and 2.2-fold, respectively. In contrast, MCF-7 cells manifested different response to Tx, inducing a twofold increase for NF-κB only without altering the ABC transporter’s genes expression levels. Treatment with U-359 led to down-regulation of the levels of all tested genes in both sensitive and resistant cells. However, the effect was much stronger in MCF-7/Tx, where U-359 down-regulated *ABCB1* and *ABCG2* gene expression of 1000 and 834 fold, respectively. Co-incubation of MCF-7/Tx cells with Tx and U-359 resulted in a substantial down-regulation of mRNA expression for all tested genes, compared to Taxol alone (Fig. 3B-D).

### Co-incubation of resistant cells with Tx and U-359 down-regulated of both ABCB1 and ABCG2

The concentration of ABCB1 and ABCG2 proteins, following the treatment with Tx, U-359 or Tx + U-359, in both MCF-7 and MCF-7/Tx cells was evaluated using human ABC ELISA kits, according to the protocol detailed in the Methods section. The results were systematically compared to the control (parental MCF-7 cells), as well as to the effects observed with Tx treatment alone in both cell lines. In MCF-7/Tx cells, a significant increase in the protein levels of both ABCB1 and ABCG2 transporters was observed. Specifically, the level of ABCB1 was elevated by 18.5%, whereas ABCG2 by 42%, compared to the control (MCF-7 cells).

**Fig. 4:**
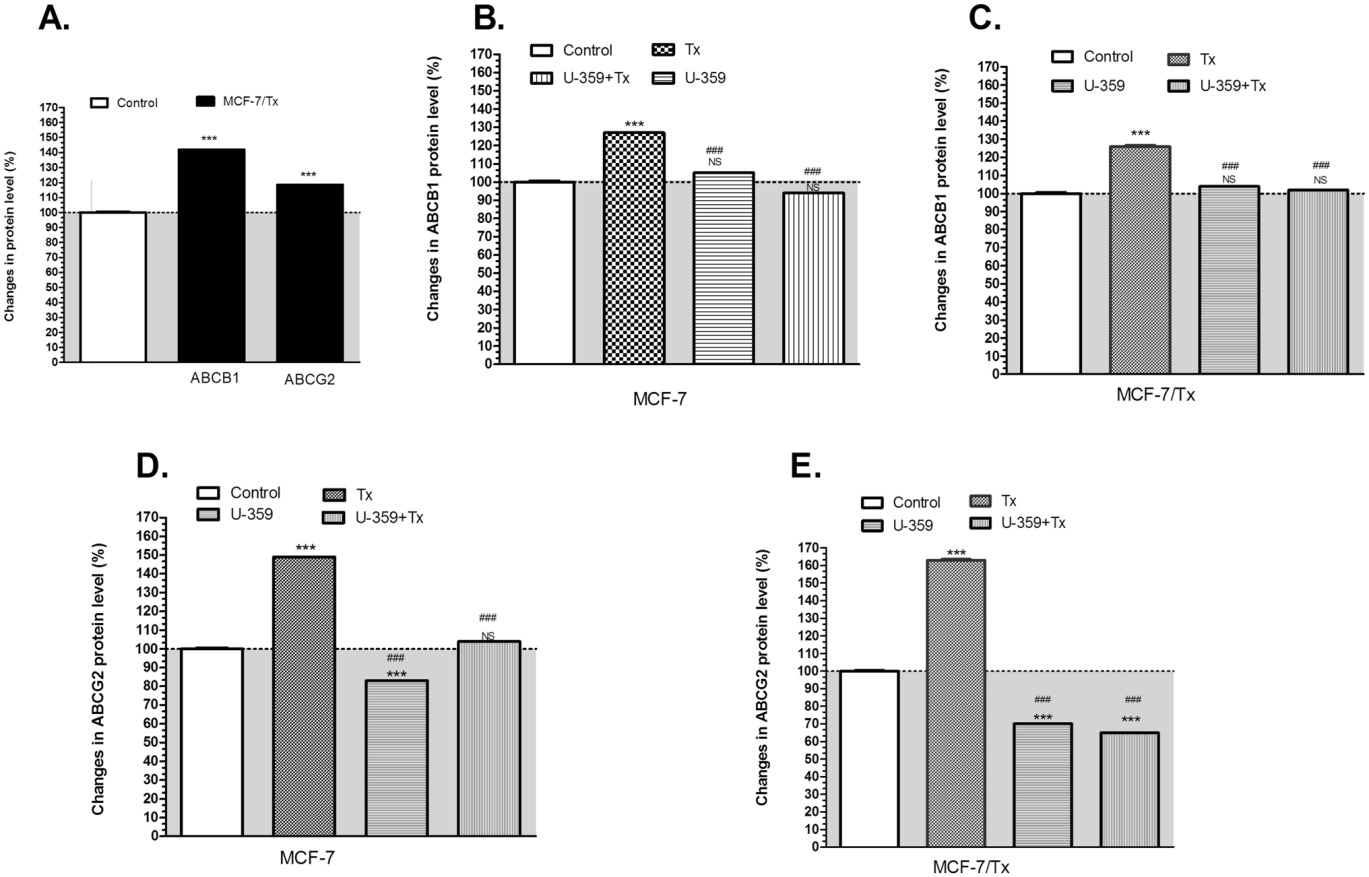
The analysis of ABCB1 and ABCG2 protein changes in MCF-7/Tx cells compared to control (parental MCF-7 cells set at 100%) (**A**). The effect of Tx, U-359, and U-359+Tx treatments (each at IC_50_ concentration) on the protein levels of ABCB1 in MCF-7 (**B**) and MCF-7/Tx (**C**) cells, and ABCG2 in MCF-7 (**D**) and MCF-7/Tx (**E**) cells. Data are presented as mean±SEM. In each experiment, three replicates were used. The control consisted of untreated MCF-7 cells. Statistical significance was assessed by one-way ANOVA and a post-hoc multiple comparison Student–Newman–Keuls test ^***^p < 0.01 in comparison with control; ^###^*p* < 0.01 in comparison with Tx

Treatment of MCF-7 cells with Tx resulted in upregulation of ABCB1 of 27% and ABCG2 of 49% in comparison to untreated MCF-7 cells (Fig. 4A, C). In MCF-7/Tx cells, Tx induced a 25% increase for ABCB1 and a 15% increase for ABCG2, compared to the untreated MCF-7/Tx cells (Fig. 4B, D). The treatment with U-359, a recognized modulator, resulted in a notable and moderate decrease in ABCG2 protein levels in both cell lines when compared to the control. On the other hand no significant changes in ABCB1 transporter expression were observed in tested cells exposed to this compound, as shown in Fig. 4C, D.

Co-incubation of resistant cells with Tx and U-359 demonstrated a remarkable down-regulation of both ABCB1 by 23% and ABCG2 by 43%, in comparison to the results obtained with Tx treatment alone. These compelling findings strongly suggest a potential synergistic effect of the combined treatment in reversing the upregulation of ABCB1 and ABCG2 observed in the resistant MCF-7/Tx cells, as clearly demonstrated in Fig. 4.

### U-359, alone or combined with Tx increased intracellular MDR dye accumulation in *cancer* cells

**Fig. 5:**
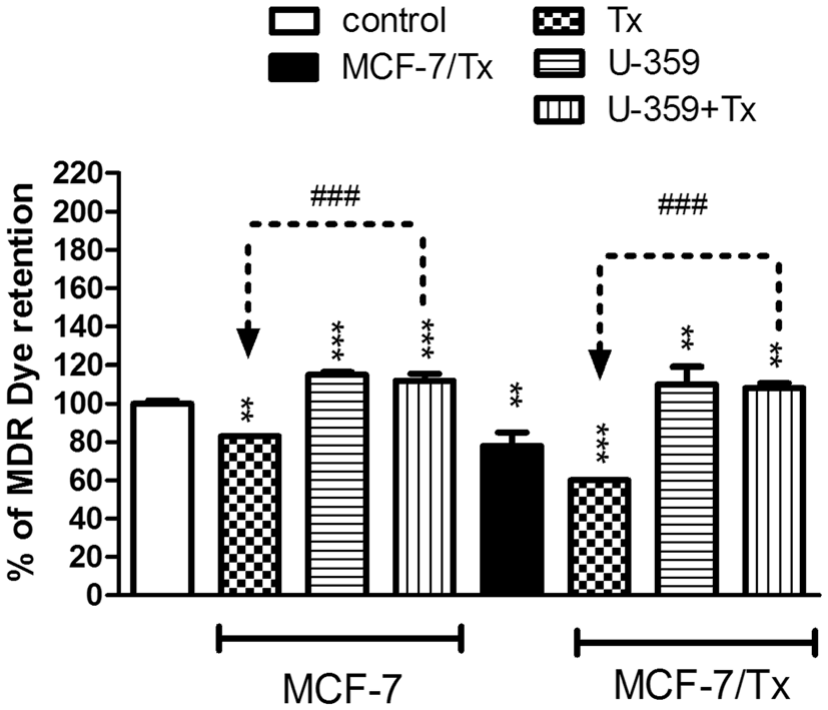
The effect of Tx, U-359 and U-359 +Tx treatments on intracellular accumulation of MDR fluorescent dye in MCF-7 and MCF-7/Tx cells. After 24 h incubation of the cells, MDR Dye Loading Solution was added to each sample and incubation was continued for additional 15 min. The fluorescence intensity was analyzed using Flexstation 3. Data are expressed as mean ± SEM. In each experiment, three replicates were used. The control consisted of untreated MCF-7 cells. Statistical significance was assessed using one-way ANOVA and a post-hoc multiple comparison Student–Newman–Keuls test. **p < 0.01, ***p < 0.001, in comparison with control; ^###^*p* < 0.001, in comparison with Tx

The fluorescent dye, acting as an indicator of ABC transporter activity, served to evaluate the potential of the tested compound as a multidrug resistance inhibitor. In cells lacking ABC transporter overexpression, the MDR dye permeates cell membranes, leading to increased fluorescence intensity (λex = 490/λem = 525 nm). Conversely, cells with the MDR phenotype expel the dye through transporters, causing a rapid decrease in fluorescence intensity, offering a method to identify MDR inhibitors. MCF-7 and MCF-7/Tx cells were treated with Tx, U-359, or U-359 + Tx (each at the IC_50_ concentration) for 24 h. As shown in Fig. 5, in MCF-7/Tx cells, the intracellular accumulation of MDR dye decreased by 22% compared to MCF-7 (control). In MCF-7 sensitive cells, exposure to Tx alone sharply reduced the intracellular accumulation of MDR dye to 83% compared with the control. Treatment of the cells with U-359 alone and in combination with Tx increased MDR dye retention in MCF-7 cells by 15% and 12%, respectively, compared to the control. In the resistant cell line, Tx significantly diminished MDR dye retention to 60%, while U-359 conversely increased it to 110%. Co-incubation of U-359 with Tx resulted in a 55% up-regulation in intracellular accumulation of MDR dye compared to Tx alone (Fig. 5).

### Combining Tx and U-359 notably reduced NF-κB p65 in both MCF-7 and MCF-7/Tx cells

**Fig. 6:**
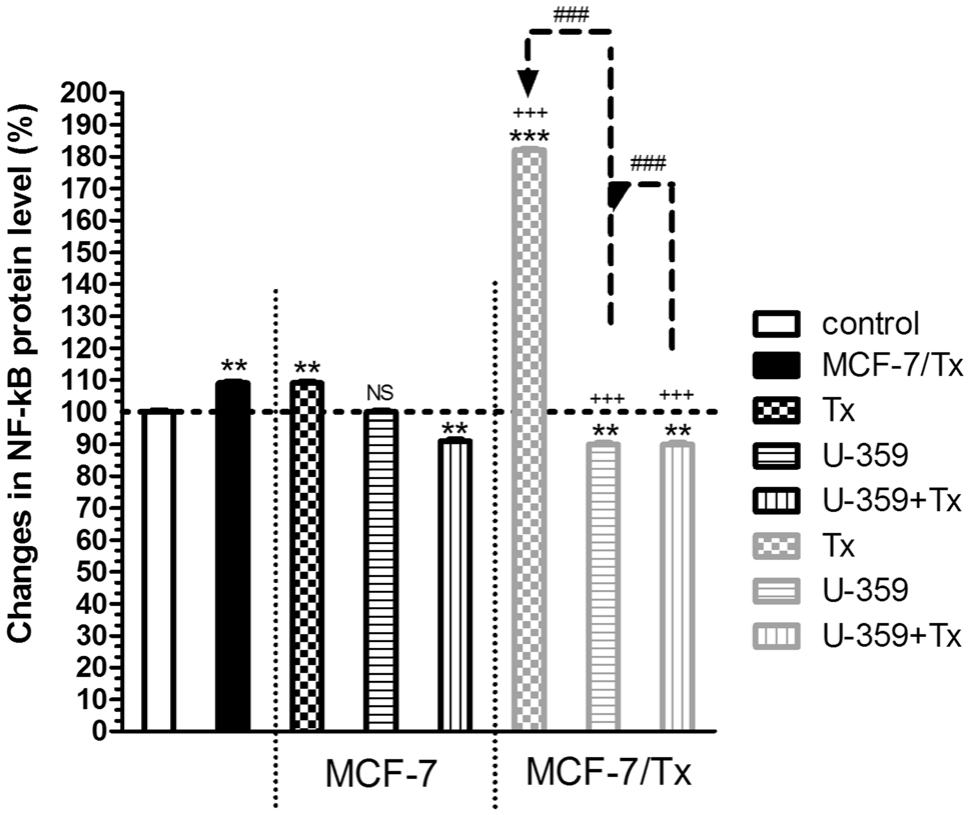
The influence of Tx, U-359 and U-359 +Tx treatments on the alteration of NF-κB protein levels in MCF-7 and MCF-7/Tx cells. The control consisted of untreated MCF-7 cells. In each experiment, three replicates were used. Results are expressed as mean±SEM, and statistical significance was determined using one-way ANOVA and a post-hoc multiple comparison Student–Newman–Keuls test, ^**^p < 0.01, ^***^p < 0.001, compared to the control, ^###^*p* < 0.001 compared to Tx; ^+++^*p* < 0.001 compared to MCF-7/Tx

The concentration of NF-κB p65 in MCF-7 and MCF-7/Tx cell lines was assessed through an ELISA-based method. Cancer cell lysates were prepared following a 24-h exposure of cells to Tx, U-359, or Tx + U-359, administered at their respective IC_50_ concentrations. Consistent with gene expression in MCF-7/Tx, there was a slight 9% up-regulation of NF-κB p65 protein level, compared to MCF-7 cells. Conversely, when MCF-7 cells were exposed to U-359, no significant changes in p65 expression were noted, while Tx increased the protein level by 9% compared to the control (Fig. 6). Co-incubation of U-359 with Tx in MCF-7 cells resulted in a 9% reduction in NF-κB/p65 protein expression compared to Tx alone. Within the resistant cell line, U-359 led to a slight reduction in NF-κB/p65 level to 90%, whereas Tx induced the opposite effect, elevating it to 167% compared to the control (Fig. 6). However, in resistant cells, the combination treatment resulted in a substantial 50% reduction in NF-κB/p65 protein level compared to the results for Tx alone. These observations suggest distinct effects of U-359 and Tx on NF-κB/p65 expression in MCF-7 and MCF-7/Tx cell lines, emphasizing the potential role of these compounds in modulating NF-κB signaling pathways.

## Discussion

Resistance to chemotherapy of cancer cells may be caused by inherent drug resistance or may develop in a response to treatment. Among different factors, the activation of NF-κB and the overexpression of ABC transmembrane proteins have emerged as crucial contributors to tumor cell resistance against chemotherapy (Ferreira et al. 2017; Fan et al. 2018; Li et al. 2019). Several chemotherapeutic agents, such as Taxol, etoposide, or doxorubicin, have been reported to activate both ABC transporters and NF-κB. In several cancer cell lines, inhibiting either NF-κB or ABC transporter activity has been shown to increase the intracellular accumulation of chemotherapeutic drugs (Długosz 2019; Fan et al. 2023). Understanding the intricate interplay between these molecular mechanisms provides valuable insights into potential strategies for overcoming drug resistance.

Over the years, extensive research has been dedicated to the discovery of substances that can inhibit ABC transporters, a group of membrane proteins critical to drug removal from cells. Inhibitors of ABC transporters can affect these proteins through multiple mechanisms, including direct interactions, modulation of intracellular ATP, and alteration of membrane phospholipids to enhance membrane permeability for ions, thereby reducing transporter activity (Wilkens 2015). The ABC transporter inhibitors can be categorized into different generations, each with distinct characteristics.

The first generation of inhibitors, including verapamil, calmodulin antagonists, and indole alkaloids, demonstrated low effectiveness. As a result, high doses were necessary, which led to increased toxicity levels. Additionally, many of these first-generation inhibitors (e.g., verapamil and cyclosporine A) are substrates for ABC transporters (e.g., ABCB1). Consequently, the use of high doses of these chemosensitizers to inhibit ABC transporter activity inevitably caused unwanted toxicities. (Hee Choi and Yu 2014; Mruk et al. 2011; Hlavata et al. 2012). Subsequent progress in inhibitor development led to the emergence of **the second generation**, featuring compounds like cyclosporin A, GF120918 (elacridar), and dexverapamil. In spite of reduced side effects of these compounds, their low affinity for ABC transporters and susceptibility to metabolism by cytochrome P450 presented limitations (Amin 2013). A new wave of ABC transporter inhibitors, known as third-generation inhibitors, has emerged through the application of quantitative structure–activity relationships (QSAR) analysis and combinatorial chemistry, exhibiting high selectivity and potency. Notable examples include tariquidar (XR9576), zosuquidar (LY335979), laniquidar (R101933), and elacridar (F12091) (Kelly et al. 2011; Durante et al. 2017; Hee Choi and Yu 2014). Despite their pharmacological promise, these inhibitors have faced limitations in clinical application due to unexpected toxic side effects and a lack of significant improvement in patient mortality during clinical trials. Nevertheless, these setbacks have not deterred the pursuit of novel P-gp inhibitors. Tariquidar, a prototype of third-generation P-gp inhibitors, has shown the capability to reverse resistance to drugs like doxorubicin and vinblastine in advanced breast cancer (Durante et al. 2017). However, its high toxicity in a phase III clinical trial for non-small cell lung cancer (NSCLC) and its susceptibility to hydrolysis (Nobili et al. 2006) led to the development of numerous tariquidar analogs to optimize its pharmacological properties. Recent studies have identified tariquidar derivatives that can reverse both ABCB1 and ABCG2-mediated drug efflux, likely through the inhibition of ATP hydrolysis, although this mechanism requires further verification via ATPase assays (Teodori et al. 2017; Antoni et al. 2020).

It is widely recognized that the upregulation of ABC transporters correlates with the activation of NF-κB (Fan et al. 2023; Abdin et al. 2021). Concurrently, the role of NF-κB in cancer initiation and progression has prompted researchers to explore NF-κB inhibitors as a complementary strategy to overcome drug resistance. Several studies have demonstrated that inhibition of ABC transporter activity using NF-κB inhibitors enhances the intracellular accumulation of chemotherapeutic drugs (Abdin et al. 2021; Rasmi et al. 2020). The combination of anticancer drugs with both NF-κB and ABC transporter inhibitors has been shown as a promised strategy in sensitizing cancer cells to chemotherapy, providing a multifaceted approach to tackle resistance mechanisms.

In this report we tested U-359, a novel synthetic 5-methylidenedihydrouracil analog which significantly inhibited proliferation in MCF-7 and MCF-7/Tx cells, as potential expression modulator of the ABCB1, ABCG2 and/or NF-κB proteins, considered for anticancer drug insensitivity in breast cancer cells. The establishment of a Taxol-resistant breast cancer cell line (MCF-7/Tx) served as a foundation for understanding the mechanisms underlying chemotherapeutic resistance.

The Tx-resistant subline was generated from the MCF-7 cell line through conventional intermittent and continuous exposure to Tx. The morphological changes observed in MCF-7/Tx cells, including larger dimensions, irregular shape, and increased nucleoli, indicated altered cellular characteristics associated with drug resistance. Additionally, the calculated resistance index (R) indicated that MCF-7/Tx cells were approximately 24-fold more resistant to Taxol than the sensitive MCF-7 cells.

Consistent with gene expression in MCF-7/Tx cells, relative ABCB1 and ABCG2 protein levels were significantly higher than in sensitive MCF-7 cells. Upon treatment with Tx, both gene and protein expression levels of transmembrane transporters were up-regulated, with ABCG2 showing the highest increase. Analysis of ABCB1 and ABCG2 gene expression profiles in MCF-7 and MCF-7/Tx cells treated with U-359 revealed a moderate down-regulation of these genes. Moreover, U-359 treatment exhibited a notable decrease in ABCG2 protein levels in both cell lines. When used in combination with Tx, U-359 significantly enhanced the treatment effect, resulting in a substantial down-regulation of both ABCB1 and ABCG2 protein levels in both cell lines, as compared to Tx treatment alone.

Tx reduced the intracellular accumulation of the fluorescent dye, a marker of ABC transporter activity, whereas U-359 increased the dye accumulation, compared to the control. Additionally, the simultaneous administration of U-359 with Tx led to a rapid augmentation in the intracellular accumulation of the MDR dye, suggesting its potential as an MDR inhibitor. These observations indicate that U-359 may function as a modulator/inhibitor of ABCB1 and ABCG2 in both MCF-7 and MCF-7/Tx cells.

To investigate the potential role of U-359 as a modulator or inhibitor of NF-κB, the influence of new compound on NF-κB level was evaluated. The quantitative detection of NF-κB/p65 protein levels revealed distinct effects of U-359 and Tx on NF-κB signaling pathways in both cell lines. U-359 showed a modest reduction in NF-κB/p65 expression, while Tx increased its levels. Importantly, the combination treatment led to a substantial reduction in NF-κB/p65 protein levels in Tx-resistant breast cancer cells, suggesting a potential role in modulating NF-κB activity to overcome drug resistance.

## Conclusion

In conclusion, our study emphasizes the critical role of ABC transporters and NF-κB in mediating resistance to chemotherapy in breast cancer cells. The interplay between these molecular mechanisms underscores the complexity of drug resistance, prompting the exploration of multifaceted strategies that would allow to overcome this problem.

Importantly, our findings reveal that U-359 reversed Tx-induced up-regulation of ABCB1, ABCG2 and NF-κB protein levels, suggesting its potential as a modulator of these proteins. Furthermore, the study indicates that U-359 may function as an MDR inhibitor, as evidenced by its ability to enhance intracellular accumulation and mitigate the efflux of the MDR dye. This dual modulatory role of U-359 on both ABC transporters and NF-κB strengthens its potential as a valuable candidate for overcoming drug resistance in breast cancer cells.

## Data Availability

All materials are available.
